# Phytomedical Properties of *Carica papaya* for Boosting Human Immunity Against Viral Infections

**DOI:** 10.3390/v17020271

**Published:** 2025-02-16

**Authors:** Rashmi Srivastava, Neeshma Jaiswal, Harsha Kharkwal, Neeraj Kumar Dubey, Rakesh Srivastava

**Affiliations:** 1 School of Life Sciences, Babasaheb Bhimrao Ambedkar University, Lucknow 226025, Uttar Pradesh, India; 2 Amity Institute of Phytochemistry and Phytomedicine, Amity University, Noida 201313, Uttar Pradesh, India; 3 Botany Department, Rashtriya PG College, Jaunpur 222003, Uttar Pradesh, India; 4 Research and Development, Helix Biosciences, New Delhi 110028, Delhi, India

**Keywords:** papaya, secondary metabolite, natural treatments, human immunity, antiviral response

## Abstract

*Carica papaya*, a tropical fruit-bearing plant, has attracted significant attention for its diverse phytomedical properties and its ability to regulate both innate and adaptive immunity, making it a promising natural therapeutic agent. *C. papaya* is rich in bioactive compounds that play a multifaceted role in immunomodulation. These bioactive constituents have demonstrated efficacy not only against the dengue virus but also against other viral infections, including COVID-19 (Corona Virus Disease 2019), Human Immunodeficiency Virus (HIV), Zika virus, and others. The antiviral effects of *C. papaya* are achieved through its ability to enhance host immunity, mitigate inflammation, reduce oxidative stress, inhibit viral replication, and modulate immune responses. These mechanisms highlight its potential as a candidate for antiviral therapies, paving the way for further exploration of its pharmacological applications and promoting eco-friendly, accessible healthcare solutions for combating viral diseases. This review highlights the antiviral potential of *C. papaya* extracts in inhibiting viral replication and modulating immune responses, emphasizing the need for further studies and clinical trials to validate their efficacy against other medically significant viruses causing human diseases.

## 1. Introduction

Viral infections, a major cause of human diseases, are intensified by globalization and increased travel, highlighting the need for effective preventive measures [1,2,3]. Viral infections pose a major global health threat, ranging from mild colds to severe diseases like HIV/AIDS and emerging zoonotic infections. Viruses contribute significantly to global morbidity and mortality, placing a substantial burden on healthcare systems and economies [4]. The impact of viral infections extends beyond individual health such as COVID-19 outbreaks and has demonstrated how rapidly viruses can spread and disrupt societies, leading to increased hospitalizations, loss of productivity, and long-term health complications like post-viral syndromes [5]. Epidemiological measures, such as incidence and prevalence rates, help assess their spread, with diseases like influenza, dengue, and chronic infections like HIV remaining major concerns. Despite advances in medication and vaccination, many viruses still lack effective treatments or vaccines. The emergence of viral escape mutants, driven by high mutation rates, enables viruses to evade immune responses, reducing the effectiveness of natural immunity and vaccines. Additionally, mutations can alter viral target sites, decreasing the efficacy of antiviral drugs [2,6]. This emphasizes the critical need for new antiviral medications, with natural compounds offering a promising avenue for discovery.

Natural compounds offer a promising avenue for such discoveries, as their diverse bioactive properties can target multiple stages of viral infection, including viral entry, replication, and immune modulation [7,8,9]. Natural compounds are characterized into primary and secondary metabolites based on their biological function, biosynthetic pathway, or source [10,11]. Primary metabolites, such as carbohydrates, lipids, and amino acids, play essential roles in cellular metabolism and can be involved in viral replication or host–virus interactions. Secondary metabolites, such as alkaloids, flavonoids, phenolics, terpenoids, lignans, saponins, etc., exhibit antimicrobial, anticancer, antidiabetic, immunomodulatory, antioxidant, anti-inflammatory, and antiviral activities. These secondary metabolites exhibit diverse bioactivities that affect multiple stages of the viral lifecycle [8,9,11,12]. Based on biosynthetic pathways, plant secondary metabolites can be broadly divided into four major categories: (i) nitrogen-containing compounds, such as alkaloids and cyanogenic glycosides; (ii) terpenes and steroids, which primarily comprise carbon and hydrogen and include flavones, flavonoids, lignin, isoorientin, tannins, and glyceollin; (iii) phenolic compounds, which include simple sugars and benzene rings; and (iv) sulfur-containing compounds, such as phytoalexins and glucosinolates. Secondary metabolites from plants play crucial roles in disease dynamics, both in terms of disease prevention and treatment, as well as in disease mechanisms, which can help uncover drug discovery opportunities [7,10,13,14,15,16,17].

Nature blesses us with many useful plant products relating to their sustainable use, be it their fruits, leaves, stems, roots, seeds, or other parts. One such treasure is the economically important plant *Carica papaya*, commonly known as papaya, classified under the order Violales, family *Caricaceae*, and genus *Carica L.*, which is a tropical evergreen tree that produces edible fruit year-round [18]. *C. papaya* likely originated in southern Mexico and Costa Rica before being brought as a plantation crop to tropical and subtropical regions worldwide [18]. *C. papaya* is a significant source of bioactive secondary metabolites with a wide range of therapeutic properties. These compounds, derived from the fruit, leaves, seeds, roots, and latex of the plant, play a vital role in treating and preventing various diseases. Accumulating pieces of evidence highlight the antiviral properties of bioactive compounds found in the papaya plant, including flavonoids, phenols, and enzymes derived from its raw and ripe fruits, leaves, and other parts [19,20,21]. While *C. papaya* extracts have gained significant attention for their inhibitory effects on the dengue virus, this review provides a comprehensive analysis of their potential as antiviral agents against various human viral infections. By compiling existing research, we highlight the antiviral properties of *C. papaya*, emphasizing its role in combating viral diseases and its potential therapeutic applications.

## 2. Insight into Secondary Metabolites and Bioactive Compounds in Different Parts of Papaya

**Figure 1:**
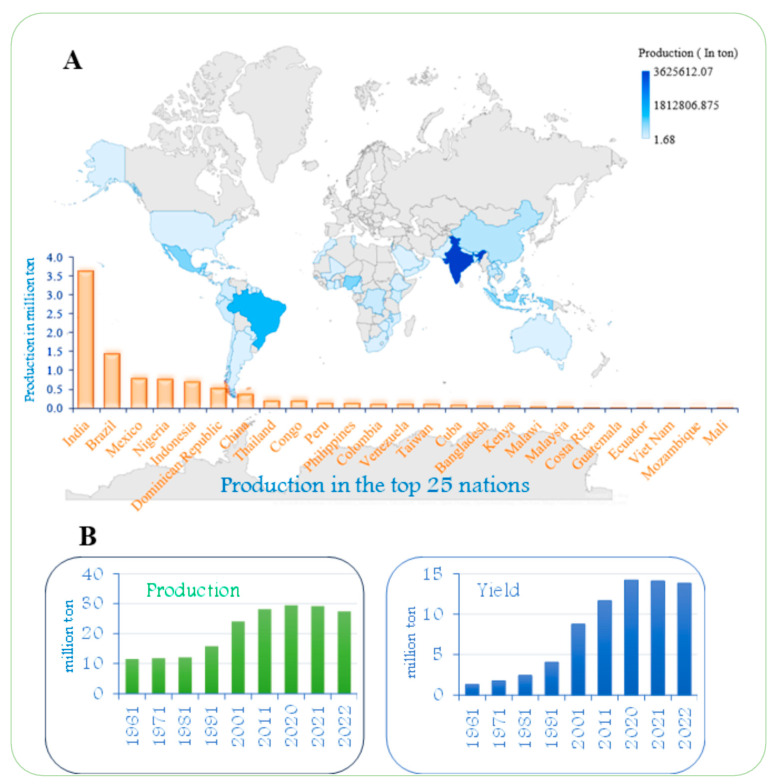
(**A**). Production of papaya in the top twenty-five countries. (**B**). Papaya production and yield in some of the top-producing countries from 1961 to 2022 (FAOSTAT; http://faostat.fao.org/, accessed on 15 December 2024).

The focus on the bioactive components of *C. papaya* is highly relevant, as the plant shows significant potential for managing viral symptoms, which is a major global public health concern in the absence of specific antiviral treatments. Papaya production flourishes in tropical regions with warm climates, as high temperatures and ample sunlight create ideal conditions for its growth. Countries such as India, Brazil, Mexico, Nigeria, and Indonesia are among the leading producers, where papaya serves as an economical crop (Figure 1). Papaya production has significantly increased over the past two decades, driven by rising demand, improved cultivation practices, and favorable growing conditions in tropical and subtropical regions (Figure 1).

*C. papaya* is primarily consumed in its ripe and raw fruit forms, but its flowers, leaves, seeds, and roots are also highly valued for their rich nutritional content, secondary metabolites, and medicinal properties (Table 1). For centuries, *C. papaya* extract has been used in traditional medicine due to its potent antioxidant properties and the presence of plant secondary metabolites such as papain, flavonoids, glycosides, and alkaloids [22]. These compounds are known for their therapeutic effects, including the treatment of malaria fever and for immune system enhancement. Additionally, papaya is rich in antioxidants like vitamins A, C, and E, as well as essential minerals [23,24]. Papain, chymopapain (A and B), endopeptidase papain III and IV, glutamine cyclotransferase, peptidase A and B, and lysozymes are among the enzymes found in papaya that have important digestive and medicinal properties. Papain is a proteolytic enzyme that helps break down proteins, improves the absorption of nutrients, promotes the formation of platelets, and is used to treat wounds and reduce inflammation. In a similar way, chymopapain lowers inflammation and treats digestive issues [25,26].

Recent studies have highlighted the phytochemical richness of *C. papaya* seeds. It is reported that the seeds contain significant quantities of alkaloids, moderate levels of glycosides, and low concentrations of resins, carbohydrates, lipids, and fixed oils [27,28]. Another work detected alkaloids, flavonoids, saponins, and phenols in the seed extracts, while subsequent work confirmed the presence of terpenoids [29,30]. The phytochemical profile extends to unripe fruit seed extracts, which contain significant levels of triterpenes and saponins [31]. It has been repeatedly shown that benzyl isothiocyanate, a sulfurous compound, is a prominent component in *C. papaya* seed extracts from several cultivars [32,33].

Assessments of *C. papaya* leaf extracts have consistently identified a broad spectrum of bioactive compounds, such as alkaloids, tripenoids, glycosides, phytosterols, and phenolic compounds. The efficiency of phytochemical extraction is highly influenced by the choice of solvent, with ethanol, methanol, ethyl alcohol, and acetone showing superior performance in extracting compounds like saponins, cardioactive glycosides, proteins, amino acids, flavonoids, phenols, tannins, and triterpenoids, highlighting their significant presence among the metabolites [31,34,35,36]. HPTLC of papaya leaf aqueous extract detected caffeic acid, myricetin, kaempferol, and trans-ferulic acid [37]. Further, the qualitative analysis of phenolics and flavonoids in *C. papaya* leaf extracts by UPLC-qTOF/MS characterized twenty-four metabolites, including two alkaloids, six phenolics, and sixteen hydroxycinnamic acid variants, and revealed elevated levels of flavonoids and phenolics surpassing those reported in earlier studies [37]. Another report revealed that major compounds identified in *C. papaya* leaves also include rutin, papain, carpaine, clitorin, and manghaslin [38]. A recent HR-ESI–MS analysis showed that *C. papaya* leaf extract contains 25% alkaloids, 15% aliphatic compounds, 20% phenolic compounds, 10% lipids, 5% glycosides, 20% terpenes, and 5% other bioactive compounds [39].

*C. papaya* pulp extract has been shown to contain a diverse range of phytochemicals, including alkaloids, terpenoids, phenolic compounds, tannins, saponins, glucosinolate, sinigrin, phlobatannins, benzyl isothiocyanate, and flavonoids [28,40]. Additionally, the fruit is rich in carotenoids such as β-cryptoxanthin, β-carotene, and lycopene, with their concentrations increasing during the ripening process. Lycopene, the pigment responsible for the red color, is found in far greater quantities in the red-fleshed cultivars, whereas it is either absent or only slightly present in yellow-fleshed cultivars [18]. Moreover, studies have identified alkaloids, reducing sugars, saponins, tannins, and terpenoids in the unripe fruit extracts of *C. papaya* [35].

Interestingly, papaya latex, a milky and sticky fluid obtained from the unripe fruit, stems, and leaves, contains a variety of bioactive compounds. Papaya latex is rich in phytochemicals and proteolytic enzymes such as papain, chymopapain A and B, and papaya peptidase A. It also contains flavonoids, alkaloids, terpenoids, and tannins, which are known for their broad antimicrobial properties [41,42].

Phytochemical analyses have uncovered a wide array of bioactive compounds in various parts of *C. papaya*, including the flower, peel, bark, and roots. The study found that papaya flower extracts contain secondary metabolites, including alkaloids, flavonoids (like rutin), saponins, steroids, triterpenoids, and tannins [22,43,44]. Quantitative and qualitative studies of *C. papaya* peel extracts using different solvents have revealed significant amounts of tannins, flavonoids, benzyl isothiocyanate, linoleic acid, and phenolic compounds [22,45]. Furthermore, caffeic and ferulic acids were identified as the dominant compounds in the peel [46]. According to recent reports, the bark of *C. papaya* contains considerable amounts of saponins, a lot of quinones, and lower amounts of phenolic compounds, coumarins, anthocyanins, and alkaloids [18]. Similarly, phytochemical screening of *C. papaya* roots confirmed the presence of phenolics, terpenes, sterol (Ergosta-5,22-dien-3-ol acetate), alkaloids, saponins, tannins, and flavonoids [22,47].

## 3. Mechanistic Effect of Papaya Bioactive Components on Viral Infection

To lessen the severity of viral diseases, several natural treatments, referred to as adjunct therapies, have been employed, with papaya extract being one of them. Papaya alkaloids, including carpaine and pseudocarpaine, exhibit various pharmacological effects that enhance immune responses and stimulate platelet production. Carpaine, a key alkaloid in papaya leaves, significantly contributes to their antithrombocytopenic properties, while pseudocarpaine offers additional antimicrobial and anti-inflammatory benefits [48,49,50,51]. The glycoside compounds may contribute to the plant’s medicinal effects, possibly affecting platelet function and immune response [52].

Polyphenolic compounds or flavonoids are known for their antioxidant and anti-inflammatory properties, and they also have antiviral activities. Caffeic acid, p-coumaric acid, gallic acid, and ferulic acid are phenolic compounds present in papaya, known for their potent antioxidant properties. Furthermore, their antioxidant effects contribute to supporting the immune system and minimizing inflammation. Quercetin, a flavonoid found in papaya leaf extracts, is a powerful antioxidant that reduces oxidative stress and inflammation, supports the immune system, and potentially inhibits viral replication [53,54,55]. Other flavonoids from *C. papaya* include Kaempferol and Myricetin, with notable antiviral, antioxidant, and anti-inflammatory properties [56,57,58,59]. Carotenoids, including beta-carotene, lycopene, and beta-cryptoxanthin, are phytochemicals in papaya that are responsible for its vibrant orange color and offer significant health benefits [60].

Papaya enzymes like papain and chymopapain support digestion, healing, and inflammation reduction, potentially lowering viral activity [50]. Other enzymes such as endopeptidase papain III and IV, peptidase A and B, and lysozymes show promise in antiviral effects by breaking down viral proteins and modulating immune responses [61]. Papaya also contains beneficial phytochemicals like saponins with antimicrobial effects, organic acids (like malic and citric) for flavor and preservation, and terpenoids (like alpha-pinene and limonene) with anti-inflammatory and antimicrobial properties [50,62]. Steroids in papayas enhance anti-inflammatory effects, while anthraquinones offer laxative and antimicrobial benefits, promoting bowel movements and fighting infections [24,63].

**Figure 2:**
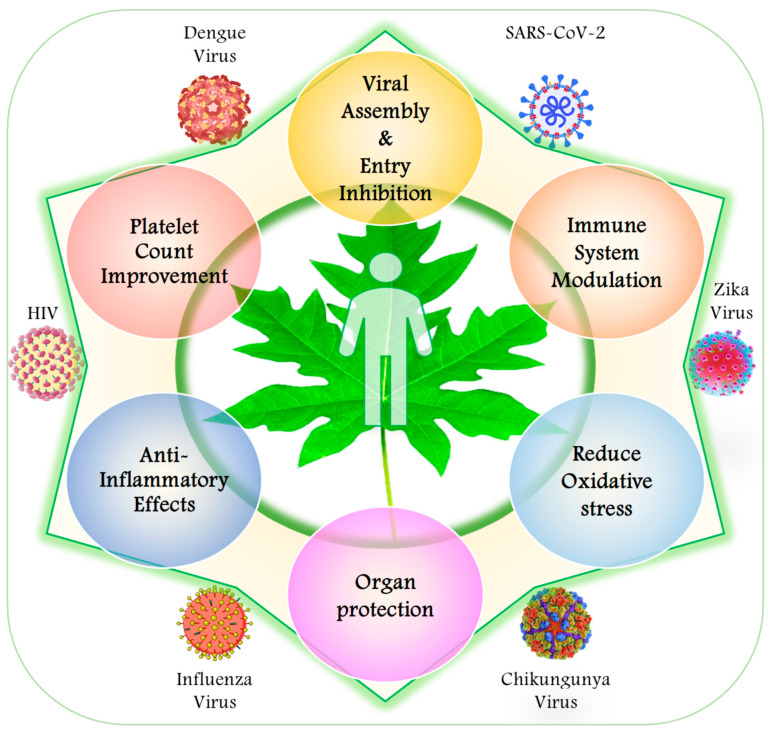
The positive consequences of papaya extract in improving human health during viral infections.

Based on accumulating reports on viral infections, the mechanisms of action of the bioactive components of papaya include the inhibition of viral assembly and entry, immune system modulation, antioxidant activity, platelet count enrichment, anti-inflammatory effects, and an organ protective effect, which could be exerted on the liver/digestive and nervous systems [64,65,66] (Figure 2; Table 1 and Table 2). The direct antiviral mechanism is of the utmost importance and involves the checking, blocking, or, more specifically, inhibition of viral entry into host cells during viral infections by interacting with viral surface proteins or by modulating host cell receptors, preventing the virus from infecting new cells [57,58,64,66,67]. The inhibition of viral replication by enzyme constituents of papaya, like papain and chymopapain, interferes with viral replication, and these enzymes can break down viral proteins, potentially hindering the virus’s ability to replicate and spread [26,68].

Another mechanistic effect of papaya bioactive components is that they enhance immune system modulation, including NK cells and T-lymphocyte activity, aiding in viral defense. They regulate the production of pro-inflammatory cytokines, such as TNF-α and IL-6, to control inflammation and prevent cytokine storms while promoting immune function for improved viral clearance and faster recovery [56]. Papaya extract has strong anti-inflammatory properties, enhances immune responses, strengthens the body’s defense mechanisms, and reduces excessive inflammation, playing a crucial role in preventing severe dengue or Zika complications like hemorrhagic fever or neurological damage [56,96,97,98]. Additionally, essential micronutrients like vitamins, enzymes (such as papain), and minerals in the extract contribute to immune support [99].

Another key mechanism of action against viruses is the antioxidant activity of papaya bioactive compounds, such as flavonoids and carotenoids, which protect host cells from oxidative damage induced by viral infections [26,99]. *C. papaya* extract has antioxidant properties that help prevent hemolysis and bleeding [100]. These antioxidants hunt free radicals, reducing cellular stress and inflammation that can support viral replication [52]. Additionally, the antioxidants in papaya leaves mitigate oxidative stress caused by viral infections, protecting cells and boosting the body’s ability to combat the virus. Antioxidant and anti-inflammatory effects also protect neural tissues, potentially alleviating virus-related microcephaly and neurological damage [52,101].

A hallmark of papaya bioactive components is their ability to modulate the platelet count by stabilizing cell membranes and preventing immune-mediated platelet destruction. This effect is particularly beneficial in viral infections like dengue and Zika, where thrombocytopenia (low platelet counts) is a common complication. *C. papaya* extract has demonstrated the ability to increase the platelet count and reduce the clotting time in thrombocytopenia, aiding recovery by stabilizing infected cell membranes and mitigating immune-related platelet destruction [64,102,103,104,105].

The bioactive components of papaya extract exhibit significant organ-protective roles in viral infections, such as mitigating Zika-induced neurological damage. Papaya’s potent antioxidant and anti-inflammatory properties protect neural tissues from oxidative stress and inflammation, potentially lowering the risk of complications like microcephaly [52,101]. Understanding the mechanisms of Papaya’s biologically active constituents can lead to sustainable approaches for preventing disease severity and supporting viral infection recovery, particularly when used alongside conventional treatments.

## 4. Effect on Emerging Viruses

Papaya exhibits promising antiviral properties by modulating immunity, inhibiting viral replication, and reducing inflammation, showing potential against emerging viruses like Dengue, Zika, and Chikungunya.

### 4.1. Anti-Dengue Viral Activity

Dengue, caused by the dengue virus and transmitted by *Aedes aegypti* mosquitoes, can lead to complications such as hemorrhagic fever, joint pain, and dengue shock syndrome, often marked by thrombocytopenia. The virus triggers inflammation, worsening tissue damage and symptoms. Over the past 50 years, dengue cases have risen 30-fold, with more than 100 million infections reported annually across more than 80 countries, mainly in tropical and subtropical regions, putting nearly 4 billion people at risk [106,107]. As there is no specific antiviral treatment, care focuses on symptom management and preventing complications [108,109]. Dengue virus, a member of the Flaviviridae family, has a very complex lifecycle and structure that allow it to infect host cells and produce a variety of clinical symptoms, ranging from a low-grade fever to severe hemorrhagic problems. The four primary serotypes of the virus are DENV1, DENV2, DENV3, and DENV4. However, people are vulnerable to reinfection because the immunity acquired from infection with one serotype does not transfer to others [110,111]. A fifth serotype, DENV5, has been identified, characterized by a sylvatic transmission cycle distinct from those of the primary serotypes [112]. Dengue virus has a positive-sense, single-stranded RNA genome (~11 kb) with a single open reading frame (ORF) that encodes a polyprotein. This polyprotein is processed into three structural proteins (C, prM, and envelope protein) and seven non-structural (NS) proteins (NS1, NS2A, NS2B, NS3, NS4A, NS4B, and NS5). Non-structural proteins are essential for viral replication, pathogenicity, and the overall viral lifecycle [113,114]. The dengue virus infects host cells via receptor-mediated endocytosis, with receptors like DC-SIGN and TIM/TAM binding to the envelope protein [115]. Inside the endosome, pH drops trigger envelope protein conformational changes, allowing fusion with the membrane and RNA release into the cytoplasm. The RNA is translated into a polyprotein, which is cleaved by proteases, while NS proteins modify ER membranes to form replication complexes. Packaged dengue genomes mature in the Golgi and are released via exocytosis [115].

Dengue virus infection triggers an immune response that can both clear the virus and worsen the disease. A key factor is antibody-dependent enhancement, where non-neutralizing antibodies from a prior infection enhance the uptake of a second serotype, increasing the disease severity. This raises the risk of dengue hemorrhagic fever or dengue shock syndrome during secondary infections. Dengue infection can also cause a cytokine storm, leading to excessive pro-inflammatory cytokines (e.g., TNF-α, IL-6, IFN-γ), which contribute to vascular leakage, plasma loss, hemorrhaging, and shock. The virus’s complex lifecycle, immune evasion strategies, and antibody-dependent enhancement make it a challenging pathogen, highlighting the need for effective vaccines, antiviral treatments, and therapeutic strategies [110,116,117,118]. *C. papaya* extract has gained attention for its potential to treat dengue fever by increasing platelet counts, reducing inflammation, and alleviating symptoms such as fever and pain, with promising results in possibly inhibiting viral replication [119].

Notably, several in silico studies have shown the promising potential of *C. papaya*, valued as both food and a quasi-drug, with its leaf extracts traditionally used to treat dengue fever. Quercetin, a flavonoid found in papaya, exhibits significant antiviral activity against DENV2 by inhibiting the NS2B-NS3 protease, which is crucial for viral replication. It binds with the protease through six hydrogen bonds, and its ADME and toxicity profiles indicate promising potential as an anti-dengue compound [57]. Another in silico study investigated seven compounds from papaya leaf extract for their potential to inhibit dengue virus NS3 and NS5 proteins. Molecular docking, MD simulations, and ADME analysis confirmed Kaempferol, Chlorogenic acid, and Quercetin as promising candidates, with Kaempferol and Quercetin showing the strongest inhibitory potential. These findings highlight the potential of these compounds as inhibitors of DENV2 NS3 and NS5 proteins, paving the way for further antiviral research [119]. Docking and MD simulations showed that 5,7-dimethoxycoumarin and *p*-coumaric acid bind stably to DENV2 NS2B-NS3 protease, with *p*-coumaric acid showing higher binding affinity [120]. Dengue NS1 protein induces thrombocytopenia via TLR4 activation and platelet aggregation. In silico analyses reveal that Rutin, Myricetin 3-rhamnoside, and Kaempferol 3-(2″-rhamnosylrutinoside) from papaya leaf extracts bind to NS1’s key residue ASN130, potentially disrupting NS1-TLR4 interaction and alleviating thrombocytopenia [121]. A docking study of nine phyto-constituents, including carpaine, dehydrocarpaine I and II, cardenolide, p-coumaric acid, chlorogenic acid, caricaxanthin, violaxanthin, and zeaxanthin, showed their potential to bind DENV3 RNA-dependent RNA polymerase, supporting papaya’s role in dengue management and its antiviral potential [69].

In vitro studies show that papaya leaf extracts can inhibit dengue virus infectivity in cultured cells [70,122]. Animal models treated with papaya leaf extract had lower virus loads and higher survival rates after dengue infection [70]. Although direct antiviral activity is challenging to confirm in human studies, patients treated with papaya leaf extract during dengue infections have shown faster recovery and reduced symptom severity, suggesting potential indirect antiviral effects [123,124,125]. Carpaine and phenolic acids are found in the leaf extract that lowers viral load by directly or indirectly influencing viral particles or host cell susceptibility [70,122]. Another study demonstrated aqueous *C. papaya* extracts significantly reduced dengue foci formation in Vero cells, as shown by a foci-forming unit reduction assay. The MTT assay confirmed cytotoxicity against virus-infected cells, with an IC50 of 137.6 µg/mL and a selective index of 75.85 [126].

Thrombocytopenia, a critical complication of dengue, is marked by a significant drop in platelet count, leading to hemorrhaging and increased bleeding risk [127]. *C. papaya* leaf extract is notable for its ability to increase platelet counts in dengue patients. Studies have shown it promotes thrombopoiesis by stimulating the bone marrow and preventing platelet destruction through membrane stabilization [103,128]. In an in vitro assay, *C. papaya* fruit and leaf extracts showed significant inhibition of hemolysis, suggesting potential therapeutic effects on diseases that destabilize erythrocyte membranes, including dengue [129]. The oral administration of pure *C. papaya* leaf extract boosts platelet and RBC counts in mice without toxicity, suggesting its potential for enhancing hematopoiesis and thrombopoiesis [130]. Standardized oral *C. papaya* aqueous extract (150 mg/kg) in thrombocytopenic rats increased thrombocytes, the delayed-type hypersensitivity response, and the phagocytic index, with minimal spleen fibrosis [37]. Active compounds like papain, flavonoids, alkaloids, and glycosides contribute to modulating platelet augmentation and hemopoiesis [76,127,131].

The bioactive compounds present in *C. papaya* directly inhibit viral replication or entry. Some studies suggest that flavonoids and alkaloids block the RNA synthesis of the virus or interfere with viral enzymes [58,64]. Flavonoids like kaempferol, quercetin, and myricetin in papaya leaves have been documented to interact with viral enzymes and pathways in various viruses, suggesting potential mechanisms that might also apply to the dengue virus [56,57,58,59]. These compounds, kaempferol, quercetin, and myricetin, inhibit the dengue virus RNA-dependent RNA polymerase essential for viral replication [78,132,133]. These flavonoids alter viral envelope proteins or host cell receptors, preventing the virus from entering host cells [134,135,136]. Flavonoids in papaya extract have been reported to inhibit a protease involved in viral assembly [67]. Carpaine, the primary alkaloid in papaya leaf extract, significantly inhibits DENV2 infection and replication, demonstrating strong anti-dengue activity [137]. A study on aqueous *C. papaya* leaf extracts showed the dose-dependent inhibition of DENV2 envelope protein expression and reduced the NS1 protein levels in an in vitro analysis using THP-1 cells treated with 100 μg/mL or 200 μg/mL of the extract, highlighting its potential inhibition of viral replication of dengue virus [76]. Another recent work suggests that the methanol extract of *C. papaya* leaves, synthesized into silver nanoparticles, inhibited DENV2 replication in vitro by over 90%, with an IC50 of 9.20 µg/mL [84]. These findings suggest potential synergistic effects of various bioactive compounds in *C. papaya* leaf extract through interactions with conserved domains of the viral NS5 protein [84].

Papaya enhances immune function by activating macrophages and T cells, promoting an antiviral response. By modulating immune responses, flavonoids could help mitigate the inflammatory response associated with dengue virus infection [134,135,136]. Bioactive compounds in papaya leaves enhance immune responses by boosting interferon production and modulating cytokine release, aiding in the control of the viral load [103,138]. A study explored the potential immunomodulatory effects of freeze-dried *C. papaya* leaf juice (FCPLJ) in AG129 mice infected with DENV2. Oral treatment with FCPLJ increased the white blood cell and neutrophil counts while reducing the plasma levels of pro-inflammatory cytokines such as GM-CSF, IL-6, and MCP-1 [138]. Treatment with papaya leaf extract in dengue patients modulates infection-induced cytokine levels, reducing pro-inflammatory cytokines (TNFα, IFNγ, and IL6) and increasing the Th2 cytokine IL4. This modulation correlates with higher platelet counts, suggesting that papaya leaf extract plays a role in modulating the immune response in dengue-induced thrombocytopenia [103].

Dengue fever is associated with high levels of oxidative stress, which causes tissue damage and intensifies symptoms. Vitamin C, flavonoids (such as Quercetin and Kaempferol), and Terpenoids (such as carotenoids) are key bioactive substances found in papaya leaf extract that function as antioxidants. The extract reinforces cellular membranes, reducing the cytopathic effects of the dengue virus. Its strong antioxidant content neutralizes free radicals in the body, reducing oxidative stress, protecting cells from harm, and promoting the immune system’s response to infection, which indirectly supports antiviral activity [52,139].

Gene expression and regulation play a crucial role in controlling stress responses and managing disease conditions by modulating the production of proteins and metabolites essential for adaptation and defense [10,17,140,141,142]. A gene expression analysis revealed high levels of *Arachidonate 12-lipoxygenase* (*ALOX 12*) and *Platelet-Activating Factor Receptor (PTAFR)* in the *C. papaya* leaf juice group, which promotes a variety of platelet activities. The study concluded that *C. papaya* leaf juice effectively boosts platelet counts in dengue fever and hemorrhagic fever patients [55]. Interferons, particularly Type I interferons, play a crucial role in inhibiting virus replication in the early stages of infection by preventing negative-strand RNA accumulation. *C. papaya* leaf extracts were shown to increase *IFN-α* expression in dengue-infected and papaya leaf extract-treated THP-1 cells [76]. Another study suggests that FCPLJ modulates cytokine release during dengue infection in AG129 mice, particularly by influencing the CCL2/MCP-1 levels at the peak of viral load. FCPLJ treatment downregulated several inflammatory cytokine genes in the liver, including *CCL6/MRP-1*, *CCL8/MCP-2*, *CCL12/MCP-5*, *CCL17/TARC*, *IL1R1*, *IL1RN/IL1Ra*, *NAMPT/PBEF1*, and *PF4/CXCL4*, indicating its potential role in mitigating inflammation during infection [139]. Additionally, FCPLJ lowered the intracellular *IL-6* and viral RNA levels in the liver [138]. The administration of *C. papaya* leaf concentrate orally reduces the IL-6 and TNF-α cytokine levels in Wistar rats [37,143].

Several clinical trials and observational studies have demonstrated that *C. papaya* leaf extract can significantly increase platelet counts in dengue patients [103,144]. In thrombocytopenic rats, a high dose of mature *C. papaya* leaf concentrate significantly increased platelets by 76.5%, WBCs by 30.51%, and RBCs by 9.08% compared to the control group [145]. A study evaluated *C. papaya* leaf extract for treating dengue fever in an elderly patient. The aqueous extract improved blood markers, including increased platelet, white blood cell, and neutrophil levels, suggesting its potential effectiveness against dengue fever [82]. Another study on 228 dengue patients with dengue fever and dengue hemorrhagic fever showed that *C. papaya* leaf juice significantly increased the platelet counts compared to standard treatment [55]. The clinical trial with dengue patients showed that *C. papaya* leaf extract capsules significantly increased the platelet count, stabilized hematocrit levels, shortened hospitalization, and accelerated platelet recovery [146,147]. In pediatric dengue fever patients, the study suggested that papaya leaf extract positively impacts RBC and WBC counts, significantly increasing platelet counts in thrombocytopenia. It was well tolerated without major adverse events, indicating its potential as a valuable supportive therapy in dengue fever management in children also [104,105,148]. These clinical studies demonstrate that papaya leaf extract increases platelet counts, strengthens immunity to infections, and aids in the prevention of thrombocytopenia consequences.

### 4.2. Anti-Chikungunya Viral Activity

Chikungunya virus (CHIKV), an arthropod-borne virus of the Alphavirus genus in the Togaviridae family, is the cause of chikungunya fever. It is predominantly spread to humans by the bite of infected Aedes mosquitos, specifically *Aedes aegypti* and *Aedes albopictus*, which acquire the virus by feeding on infected hosts. CHIKV has been reported in over 100 countries across the Americas, Africa, Asia, Europe, and the Indian and Pacific Oceans. Seroprevalence data, observed cases, and mosquito distributions suggest CHIKV transmission in 104 countries, affecting 2.8 billion people. Outbreaks occur every 6.2 years, infecting 8.4% of the vulnerable population each time [149]. With 33.7 million annual infections, mainly in Southeast Asia, Africa, and the Americas, targeted vaccination or anti-CHIKV drugs could significantly reduce infections, deaths, and disability-adjusted life years [149,150].

The CHIKV contains a single-stranded positive-sense RNA genome of approximately 11.8 kb and is classified as a single serotype, offering lifetime immunity upon infection. CHIKV has an icosahedral capsid and a genome with two ORFs: four 5′-end ORFs encoding non-structural proteins (NSP1–NSP4) and five 3′-end ORFs encoding structural proteins (C-E3-E2-6K/TF-E1) [151]. Clinically, the acute phase of the disease is distinguished by a rapid high temperature, crippling joint pain and swelling, a maculopapular rash on the trunk and limbs, and other symptoms such as muscular discomfort, headache, nausea, and conjunctivitis. In certain cases, a chronic phase develops, with persistent joint discomfort that can last months or years, potentially resulting in long-term impairment and demanding management techniques such as physiotherapy and pain medication [152].

An in silico study docked four phytochemicals from *Papaya* leaf—p-coumaric acid, caricaxanthin, violaxanthin, and zeaxanthin—against chikungunya virus glycoprotein (E3-E2-E1) and non-structural protein 2 (NSP2) protease. The results indicated that violaxanthin had the best docking score against the glycoprotein, while zeaxanthin showed the highest efficacy against NSP2, suggesting the potential of papaya leaf extract in combating CHIKV [69].

In vitro studies demonstrated that the *C. papaya* leaf powder formulation significantly reduced CHIKV, as confirmed by a focus-forming unit assay post-infection. This powdered formulation exhibited anti-chikungunya activity, reducing viral titers under post-treatment and pretreatment at 100 μg/mL, with no effect in cotreatment, while the viral RNA levels remained unchanged. An immunofluorescence assay using green fluorescence in infected cells further confirmed a dose-dependent reduction (12.5–100 μg/mL) in the CHIKV antigen levels, with the highest inhibition observed at 100 μg/mL post-treatment [70]. Interestingly, the methanolic extract of *C. papaya* exhibited substantial larvicidal and pupicidal activities against the chikungunya vector, *A. aegypti* [71]. Owing to its immunomodulatory, anti-inflammatory, and platelet-boosting properties, nanoparticle-conjugated papaya leaf extract has been studied for CHIKV infections. For example, a study showed that papaya leaf-derived silver nanoparticles inhibited CHIKV by 39% at 125 μg/mL and 52% at 62.5 μg/mL, with 14% cell viability in infected Vero cells. These results suggest AgNPs as potential antiviral agents against CHIKV [72].

Papaya extract stimulates the immune system to effectively combat viral infections, confirming its immunomodulatory effects against CHIKV [153]. CHIKV infections, like dengue, can occasionally cause thrombocytopenia, in which case, papaya extract has been shown to enhance platelet production by stimulating bone marrow activity and hence improving platelet counts [70,102]. Limited publications exist on the effects of papaya leaf extract on CHIKV. Further research is needed to explore its potential antiviral and immunomodulatory properties against CHIKV-induced infections.

### 4.3. Anti-Zika Viral Activity

Zika Virus (ZIKV) is an RNA virus from the Flaviviridae family, transmitted primarily by *Aedes* mosquitoes, particularly *Aedes aegypti* and *Aedes albopictus*. In addition to mosquito bites, ZIKV can be transmitted sexually, vertically from mother to fetus, and, in rare cases, through blood transfusions. It can cause mild symptoms like fever and rash but is associated with severe complications, such as microcephaly in newborns and Guillain-Barré syndrome in adults. Since 2007, ZIKV has spread rapidly, causing outbreaks in Micronesia, the South Pacific, and the Americas. The 2015–2016 Brazilian outbreak heightened concerns due to a surge in microcephaly cases, with a mortality rate of 52.6 per 1000 person-years among affected children. ZIKV remains prevalent in Latin America and the Caribbean, with sporadic cases globally. Climate change is expected to accelerate its spread, potentially putting 1.3 billion more people at risk by 2050, particularly in Europe, North America, and temperate Asia [154,155].

ZIKV has a positive-sense RNA genome of about 10.8 kb, encoding a polyprotein cleaved into structural proteins—Capsid (C), premembrane/membrane (prM/M), and Envelope (E)—and non-structural proteins—NS1, NS2A, NS2B, NS3, NS4A, NS4B, and NS5. These proteins are essential for viral structure, replication, and immune evasion. While there is no specific antiviral treatment for ZIKV, natural remedies (including papaya) extract are being explored for their potential antiviral and immunomodulatory properties [19,101]. ZIKV and Dengue virus share a similar flavivirus structure but exhibit some key molecular differences [156,157]. The cryo-EM structure of mature ZIKV closely resembles DENV, but the most significant structural differences occur near the glycosylation site, a region linked to neurotropism. Variations in this site suggest differences in receptor binding and immune recognition. Additionally, ZIKV has a six-residue insertion in the E protein, modifying the fusion loop region, which affects membrane fusion, antibody binding, and immune evasion [158,159,160]. These structural differences likely impact viral attachment, immune response, and disease progression.

ZIKV’s neurotropism and unique pathogenic mechanisms require targeted treatments, which may not be fully addressed by papaya extract alone. However, papaya extract shows promise as a complementary therapy for ZIKV due to its antiviral, antioxidant, anti-inflammatory, and immune-boosting properties. The secondary metabolites in papaya extract inhibit viral replication, reduce inflammation, and help restore platelet counts, providing symptomatic relief and potential antiviral effects. For example, the bioactive compounds in papaya disrupt ZIKV replication by inhibiting key viral enzymes, including NS5 protease and RNA-dependent RNA polymerase, thereby suppressing viral RNA synthesis and blocking replication [66,96,97]. A study demonstrates that papaya fruit pulp inhibits ZIKV infection in A549 cells without affecting cell viability. The findings suggest that papaya pulp extract prevents ZIKV attachment to A549 cells, thereby blocking the initiation of the viral infectious cycle [96].

Further, an in silico screening study addresses the potential of papaya-derived phytochemicals as antiviral agents against ZIKV replication [19]. Seven compounds, β-sitosterol, carpaine, violaxanthin, pseudocarpaine, Δ7-avenasterols, rutin, and *cis*-β-carotene, exhibited high binding affinities to the RNA methyltransferase and RNA-dependent-RNA-polymerase (RdRp) domains of ZIKV NS5, with β-sitosterol displaying the most favorable binding energy. ADMET analysis confirmed their promising pharmacokinetic properties and non-toxic profiles, suggesting the potential of these compounds as ZIKV replication inhibitors [19]. However, experimental validation and assessments of bioavailability and safety are needed to advance these compounds as drug candidates, providing a foundation for natural antiviral therapies against ZIKV.

## 5. Effect on Respiratory Viruses

Respiratory viruses are a diverse group of pathogens that infect the respiratory system, causing illnesses from mild colds to severe conditions and spreading through respiratory droplets, direct contact, and, occasionally, airborne transmission. Accumulating research suggests that papaya and its bioactive compounds prevent or reduce the severity of respiratory viral infections, including influenza and SARS-CoV-2. While these findings indicate potential effectiveness against other respiratory viruses, further clinical studies are needed to confirm their therapeutic efficacy.

### 5.1. Anti-Influenza Viral Activity

Influenza is a seasonal and pandemic virus that infects up to 1 billion people annually, primarily in winter, with WHO estimating 3 to 5 million severe cases and 290,000 to 650,000 respiratory deaths each year [161]. Influenza viruses are highly contagious pathogens causing the flu, a respiratory illness in humans. Belonging to the Orthomyxoviridae family, they are classified into four types: A, B, C, and D. Types A and B are responsible for seasonal epidemics, with Type A further divided into subtypes based on hemagglutinin (HA) and neuraminidase (NA) surface proteins. Influenza A (e.g., H1N1 and H3N2) causes seasonal flu, while Influenza B affects only humans. Influenza C causes mild illness, and Influenza D infects cattle without harming humans. The influenza virus has a single-stranded RNA genome and a lipid envelope with HA and NA proteins essential for entry and release [162,163,164].

Papaya secondary metabolites show promising antiviral, immunomodulatory, and anti-inflammatory properties necessary for combating influenza virus infections. An in silico study suggested that chlorogenic acid effectively inhibits influenza A (H5N1) virus neuraminidase [95]. Another in silico study docked four papaya leaf secondary metabolites—p-coumaric acid, chlorogenic acid, violaxanthin, and zeaxanthin—against influenza A (H1N9) virus neuraminidase (NA), suggesting the potential of papaya leaf extract in combating influenza virus [69]. P-coumaric acid, a precursor in flavonoid biosynthesis, has shown antiviral effects against influenza in a mouse model by increasing survival rates and lowering the virus [93]. The influenza A subtype H7N9 virus exhibits high lethality in humans, with neuraminidase mutations posing significant medical challenges. Molecular docking identified quercetin, chlorogenic acid, baicalein, and oleanolic acid as promising inhibitors with stable binding to both wild-type and mutated neuraminidase, offering potential treatments for drug-resistant H7N9 strains [94]. Although secondary metabolites from various plants have shown significant effects on influenza infection [92], the potential of papaya extract remains largely unexplored for anti-influenza and requires further investigation for effectiveness.

### 5.2. Anti-SARS-CoV-2 (COVID-19) Activity

SARS-CoV-2 (Severe Acute Respiratory Syndrome Coronavirus 2) is the virus responsible for COVID-19 (COronaVIrus Disease 2019). Since the first reported case in Wuhan, China, in November 2019, COVID-19 has rapidly spread across all continents. As of January 2025, over 750 million confirmed cases have been reported worldwide [165]. It is an enveloped, positive-sense virus belonging to the Coronaviridae family and the genus Coronavirus. SARS-CoV-2 has a single-stranded RNA genome of about 30 kilobases (kb) in length, making it one of the largest RNA virus genomes [166]. The genome encodes for 29 proteins, including the structural proteins and non-structural proteins required for replication, transcription, and viral assembly. Its spike protein binds to the angiotensin-converting enzyme 2 (ACE2) receptors on host cells, primarily in the human respiratory system [167]. Although vaccines and antiviral drugs have been developed, there is still an ongoing search for natural remedies with potential complementary therapeutic benefits.

Several in silico studies have also suggested that *C. papaya* bioactive components show significant potential as an herbal antiviral agent against SARS-CoV-2 by targeting key viral proteins to disrupt its lifecycle [58,74,168]. For example, an in silico docking and molecular dynamics simulation study investigated papaya leaf extract as a potential COVID-19 therapy, targeting SARS-CoV-2 proteins like the nucleocapsid, main protease (MPro), RdRP, and spike proteins of the Wuhan, Delta, and Omicron variants. Several compounds from papaya leaf extract, such as protodioscin, clitorin, glycyrrhizic acid, manghaslin, kaempferol-3-(2g-glucosylrutinoside), rutin, isoquercetrin, and acacic acid, demonstrated strong binding affinity to these viral targets. Protodioscin exhibited the strongest binding energies with key proteins, including the nucleocapsid, MPro, RdRP, and spike protein. It effectively interacts with spike protein residues essential for ACE2 receptor binding, potentially blocking this interaction and inhibiting SARS-CoV-2 entry and fusion, offering a promising avenue for therapeutic intervention [168]. Another in silico study evaluated whether 40 phytoconstituents of papaya leaves against six SARS-CoV-2 proteins and 20 compounds met the criteria for drug-likeness, non-toxicity, and pharmacokinetics. Molecular docking against six viral targets (3-chymotrypsin-like protease (3CLpro), papain-like protease (PLpro), RdRp, endonuclease (EndoU), S1 and S2 region of spike protein) revealed that deoxyquercetin, kaempferol, catechin, and apigenin exhibited strong binding affinities to 3CLpro, S1, and EndoU. These secondary metabolites demonstrate multi-target potential, with binding affinities ranked S1 > 3CLpro > EndoU > RdRp > PLpro > S2, supporting their therapeutic promise [58]. Further, specific compounds from papaya leaf extract, such as phenol-2-methyl-5-(1,2,2-trimethylcyclopentyl)-(S)- and beta-mannofuranoside-farnesyl, have been identified as targeting key viral proteases, 3CLpro and PLpro, and demonstrated safety and effectiveness against SARS-CoV-2 [75]. A virtual screening approach revealed that 1,8-Dichloro-9,10-diphenylanthracene-9,10-diol and lupeol from papaya exhibited significant binding affinity to the SARS-CoV-2 spike glycoprotein and main protease, indicating potential inhibitory effects and protective properties against the virus [169].

A significant study using in vitro and in silico approaches assessed the antiviral potential of papaya leaf extract against SARS-CoV-2 through a crystal violet assay, MTT cytotoxicity assay, molecular docking, and modeling. The compounds present in papaya leaf extracts such as fatty acids, sterols, triterpenes, and alkaloids demonstrated strong binding to key viral targets in molecular docking and simulations. Terpenoids like 2β,3β-dihydroxy-ursolic acid inhibited RdRp, while sterols and alkaloids targeted PLpro, RBD-spike proteins, and viral replication pathways [74]. A recent study by Cao et al. (2024) highlighted the pharmaceutical potential of papaya leaves in treating inflammation and coronavirus infections [23]. Papaya leaf juice exhibited strong anti-inflammatory effects by suppressing nitric oxide production, inhibiting the iNOS and COX-2 protein levels, and reducing LPS-induced TLR4 activation via the MAPK pathway. It also demonstrated antiviral activity against beta-coronaviruses (HCoV-OC43 and SARS-CoV-2) and the alpha-coronavirus HCoV-229E. An HPLC-QTOF-MS analysis identified key phytochemicals including quercetin, kaempferol glycosides, and carpaine, with the synergistic components proving more potent than individual compounds [23]. Fermented *C. papaya* and *Morinda citrifolia* supplements are used in post-COVID recovery protocols to reduce symptoms and improve the quality of life. Clinical improvements are observed, including reductions in inflammatory markers such as the IL-6 and IL-8 levels and nitric oxide metabolites, alongside enhanced phagocytic capacity, antioxidant activity, and ATP levels [170]. These findings highlight the potential of fruit-based supplements to mitigate post-COVID symptoms through immune modulation, redox balancing, and energy restoration.

## 6. Effect on Sexually Transmitted Diseases

### 6.1. Anti-Human Immunodeficiency Virus Activity

Human Immunodeficiency Virus (HIV) is a retrovirus that causes AIDS, a condition leading to the progressive weakening of the immune system and making individuals vulnerable to infections. Since the start of the epidemic, an estimated 88.4 million people have been infected with HIV, with 42.3 million deaths. By the end of 2023, 39.9 million people were living with HIV, with an estimated 0.6% of adults aged 15–49 affected globally. The WHO African Region remains the most impacted, with 3.4% of adults living with HIV, accounting for over two-thirds of global cases [171]. HIV is classified into two main types: HIV-1, the most prevalent and virulent form worldwide, and HIV-2, which is less common and has slower disease progression. Structurally, HIV is an RNA virus enveloped in a lipid bilayer derived from the host cell. Its genome contains key genes such as *gag*, encoding structural proteins; *pol*, encoding enzymes like reverse transcriptase, integrase, and protease; and *env*, encoding envelope glycoproteins (gp120 and gp41) essential for viral entry into host cells. HIV infection progresses through distinct stages—acute infection, clinical latency, and AIDS—marked by a gradual depletion of CD4+ T cells, leading to immune system failure if untreated [172]. While no cure exists, antiretroviral therapy (ART) effectively suppresses HIV replication, allowing individuals to manage the infection and lead relatively healthy lives [173].

Thrombocytopenia is common in HIV patients, and papaya extract increases the platelet count, benefiting those with similar hematological issues [66]. It also alleviates fatigue and nutritional deficiencies due to its rich nutrient profile. While papaya extract cannot replace ART, in vitro studies are needed to evaluate its impact on HIV replication and its potential as an adjunct therapy to enhance immune health and quality of life. Complementary therapies like papaya extracts are gaining attention for their immune-boosting, antioxidant, and anti-HIV properties. By stimulating the immune response, the extract helps delay immune suppression caused by HIV and improves the body’s ability to combat infections [39]. Methanol and aqueous extracts from *C. papaya* aerial parts showed anti-HIV-1 activity due to phytoconstituents such as flavonoids, tannins, carbohydrates, triterpenes, and alkaloids [89]. Another in vitro study showed that *C. papaya* leaf extracts exhibit cytotoxicity and significant anti-HIV-1 activity and inhibit HIV-1 protease. The extracts also reduce HIV-1-induced ROS production, demonstrating antioxidant potential and further suppressing viral replication [39].

Kaempferol, quercetin, and other polyphenols are known for their antiviral properties and immune-enhancing effects. In light of this, an in silico study was conducted to evaluate the antiviral activity of *C. papaya* phytochemicals against HIV-1 protease. Molecular dynamics simulations and the Molecular Mechanics/GB Surface Area method indicated that Kaempferol-7-glucoside, Epigallocatechin, and Luteolin effectively bind to the HIV-1 protease active site, demonstrating favorable binding free energies. Further binding energy analyses highlighted Kaempferol-7-glucoside as a promising inhibitor of HIV-1 protease, with favorable structural interactions supporting its potential as a therapeutic agent [68].

### 6.2. Anti-Human Papillomavirus Activity

Human Papillomaviruses (HPVs) are a group of over 200 related viruses and some of the most prevalent sexually transmitted infections worldwide. While most HPV infections cause no harm, these small, non-enveloped, double-stranded DNA viruses belong to the Papillomaviridae family. However, certain types can lead to genital warts or increase the risk of cancer. The global prevalence of genital HPV infection is similar in men and women (3.5–45% vs. 2–44%) and is higher in developing countries (42.4%) than in developed countries (22.6%) [174]. According to Giuliano et al. (2003), a weekly dietary intake of papaya, a rich source of carotenoids, was significantly associated with a reduced risk of developing chronic HPV infection. The protective effect of papaya is attributed to its rich content of dietary carotenoids, particularly β-carotene, which support immune function through their antioxidant properties and help reduce the likelihood of persistent infections [90].

## 7. Other Antiviral Activity

Recently, the world has witnessed a rise in the incidence of viral infections and antiviral treatment resistance, emphasizing the urgent need for new therapeutic strategies. Papaya extracts, rich in bioactive compounds, hold significant potential as they target multiple phases of the viral replication cycle. This multi-target approach can prove more effective in preventing viral infections and minimizing the development of resistance. Studies have demonstrated that papaya plant extract and its bioactive components can inhibit viral replication and entry, not only in the viruses discussed above but also in several other medically significant viruses, such as herpesvirus and hepatitis viruses, showcasing their broad-spectrum antiviral potential.

*C. papaya* leaf extract has demonstrated potential antiviral activity against Herpes simplex virus 1 (HSV-1), one of the most prevalent pathogens worldwide. Studies have highlighted its effectiveness in lowering antiviral concentrations and favorable selectivity indexes, suggesting that it is a promising alternative for combating resistant strains of the virus due to its notable anti-herpetic activity [87]. Apigenin-containing papaya leaf extract is used to enhance immunity in dengue [88]. In an in silico assessment, molecular docking results revealed that apigenin demonstrated superior docking scores, binding affinities, and MM-GBSA scores when interacting with the gamma herpesvirus cyclin-CDK complex. Pharmacophore and pharmacokinetic evaluations further highlighted its promising pharmacological potential. These findings suggest that apigenin could serve as a potential anti-herpes agent, though further in vitro and in vivo validation is required [88].

Hepatitis C virus (HCV) is a blood-borne virus that causes hepatitis C, a liver infection that can vary from mild to severe, potentially leading to chronic liver disease or even liver failure. Patients with HCV-related cirrhosis are often ineligible for antiviral treatment, making adjunctive therapies crucial. Oxidative DNA damage is an early event in HCV infection. A study tested a fermented papaya preparation (FPP) at 9 g/day for six months in such patients. The FPP significantly reduced oxidative DNA damage as measured by 8-hydroxy-deoxyguanidine (8-OHdG), which serves as a molecular marker of oxidative stress, and is associated with pathological conditions. The FPP also significantly improved the cytokine balance by lowering pro-inflammatory cytokines TNF-α and TNFR2 (Tumor Necrosis Factor Receptor 2) levels. These findings suggest the FPP’s potential as a supportive antioxidant/immunomodulator therapy for HCV-related cirrhosis [86].

However, these initial studies on viruses such as HPV, herpesvirus, and hepatitis viruses suggest that papaya extract may impact viral infections beyond those mentioned above. Further research is needed to confirm these findings, elucidate the underlying mechanisms, and explore their effects on a broader range of viruses.

## 8. Limitations and Challenges

Despite the promising antiviral potential of *C. papaya*, several challenges limit its widespread use and clinical application. These include scientific and technical barriers, as well as regulatory and safety concerns. The concentration of secondary metabolites in papaya extracts varies depending on factors like the plant’s growth conditions, maturity stage, and extraction methods, complicating standardization for therapeutic use. Establishing an effective dosage is difficult due to inconsistencies in active ingredient concentrations. While papaya exhibits antiviral activity, the exact molecular mechanisms of many secondary metabolites remain unclear, hindering targeted drug development. Many secondary metabolites, such as flavonoids and phenolic compounds, have low bioavailability due to poor solubility or rapid metabolism, while papaya extracts may degrade over time, losing their bioactive potential during storage or transport. High doses of papaya extracts, containing papain or carpaine, cause toxicity, gastrointestinal irritation, or cardiovascular effects. Additionally, papaya latex can trigger allergic reactions, and unripe papaya or its latex induces uterine contractions, posing risks for pregnant women.

Most studies focus on dengue, with limited exploration of its efficacy against other medically important viruses like HIV, SARS-CoV-2, Zika, chikungunya, or influenza (Table 1). Although research suggests papaya extract has antiviral properties, most studies are still in the early stages, and more clinical trials are needed to validate its effectiveness. Papaya’s antiviral properties may be specific to certain viral strains, limiting its use as a broad-spectrum treatment.

Establishing papaya-based bioactive compounds as potent antivirals will require rigorous, time-consuming, and costly testing. Despite its widespread use in traditional medicine, transitioning to scientifically validated pharmaceutical applications faces cultural and technological gaps. Increased demand for papaya-based products could lead to overharvesting, affecting the plant’s sustainability and bioactive compositions. To address these challenges, standardized protocols for cultivating, harvesting, and processing papaya are essential, as well as investments in clinical trials to validate its safety and efficacy. Involving experts from pharmacology, traditional medicine, and biotechnology will be crucial in integrating papaya into modern therapeutic frameworks.

The transition to clinical application requires overcoming issues related to standardization, bioavailability, toxicity, and regulatory approval. Therefore, comprehensive research, including mechanistic studies, formulation optimization, and clinical trials, will be essential to fully harness the therapeutic potential of papaya. Addressing these challenges will enable *C. papaya* to become a valuable tool in the fight against viral diseases, offering a sustainable and accessible solution to global health challenges. Its use as a natural, plant-based antiviral agent highlights the importance of biodiversity and traditional knowledge in modern drug discovery.

## 9. Conclusions

*C. papaya* represents a promising natural source of antiviral agents due to its rich reservoir of bioactive secondary metabolites, including flavonoids, phenolic compounds, alkaloids, and proteolytic enzymes. While these secondary metabolites offer immense therapeutic potential, understanding their dual roles in both healing and harm is critical for their effective utilization in medical science, leading to innovative treatments for some of the most challenging diseases. These compounds exhibit multifaceted antiviral mechanisms, such as inhibiting viral replication, blocking viral entry, modulating host immunity, mitigating inflammation, minimizing the risk of resistance development, and being used as adjunct therapies. By harnessing these unique attributes, natural compounds hold significant potential for addressing current challenges in managing viral infections, including those caused by emerging and re-emerging viruses. It should be specifically mentioned again that the papaya plant has shown significant potential in managing dengue virus infections and holds promise against other medically important viruses, such as chikungunya, SARS-CoV-2, Zika, HIV, and influenza. Further research is needed to explore the effectiveness of papaya extract against other viral infections that impact human health, such as hepatitis, herpes, and other viruses.

## Figures and Tables

**Table 1:** Function of bioactive compounds present in *Carica papaya* against viral infections in humans.

Bioactive Compound #	Category	Plant Part	Activity/Function
**ALKALOIDS**			
Carpaine	Alkaloid	Leaf, seeds	Anti-thrombocytopenia, enhances immunity
Pseudocarpaine	Alkaloid	Leaf	Anti-thrombocytopenia, antimicrobial and anti-inflammatory
Dehydrocarpaine I and II	Alkaloid	Leaf	Anti-hypertensive, antimicrobial properties
**PHENOLIC COMPOUND AND FLAVONOIDS**			
P-Coumaric Acid	Phenolic Compound	Leaf	Antioxidant, anti-inflammatory, antiviral potential
Chlorogenic Acid	Phenolic Compound	Leaf, seeds	Antioxidant, antiviral potential
Gallic acid	Phenolic Compound	Fruit peel, leaf	Antioxidant, antiviral potential
Ferulic acid	Phenolic Compound	Fruit peel, leaf	Antioxidant, antiviral potential
Caffeic Acid	Phenolic Compound	Leaf, fruit peel	Antioxidant
Baicalein	Flavonoid	Leaf	Antioxidant, anti-inflammatory
Quercetin	Flavonoid	Leaf, fruit	Antioxidant, antiviral potential, anti-inflammatory
Rutin	Flavonoid	Leaf, flower	Antioxidant, antiviral potential, anti-thrombocytopenia
Myricetin	Flavonoid	Leaf	Antioxidant, antimicrobial, anti-thrombocytopenia
Apigenin	Flavonoid	Leaf	Antiviral potential, enhances immunity
Kaempferol	Flavonoid	Leaf, fruit	Antiviral potential, antioxidant, anti-inflammatory
Kaempferol 3-(2″-rhamnosylrutinoside)	Flavonoid Glycoside	Leaf	Antioxidant, anti-inflammatory, anti-thrombocytopenia
Kaempferol-7-glucoside	Flavonoid Glycoside	Leaf	Enhances immunity, antiviral potential
Catechin	Flavonoid	Leaf	Enhances immunity, antiviral potential
Epigallocatechin	Flavonoid	Leaf	Antiviral potential
Deoxyquercetin	Flavonoid	Leaf	Antiviral potential
Luteolin	Flavonoid	Leaf	Antiviral potential
5,7-Dimethoxycoumarin	Coumarin	Leaf	Antiviral potential
Quinones	Phenolic Compound	Bark, leaf	Antimicrobial, antioxidant
Tannins	Polyphenol	Leaf, seeds, pulp, unripe fruit, latex, root	Antimicrobial, defense mechanism
Phlobatannins	Phenolic Compound	Leaf, pulp	Anti-inflammatory
Anthraquinones	Phenolic Compound	Leaf	Antimicrobial
Phenol-2-Methyl-5-(1,2,2-Trimethylcyclopentyl)-(S)-	Phenolic Compound	Leaf	Antiviral potential
**CAROTENOIDS**			
Caricaxanthin	Carotenoid	Leaf	Antiviral potential
Violaxanthin	Carotenoid	Leaf	Antiviral potential
Zeaxanthin	Carotenoid	Leaf	Antiviral potential
β-Carotene	Carotenoid	Fruit, leaf	Antiviral potential
β-Cryptoxanthin	Carotenoid	Fruit, leaf	Antiviral potential
Cis-β-Carotene	Carotenoid	Leaf	Antiviral potential
**GLUCOSINOLATES AND SULFUR COMPOUNDS**			
Sinigrin	Glucosinolate	Fruit pulp	Antimicrobial
Benzyl Isothiocyanate	Isothiocyanate	Seeds, leaf, pulp	Antimicrobial
**TERPENOIDS, STEROLS AND TRITERPENOIDS**			
α-Pinene	Monoterpene (Terpenoid)	Seeds, fruit	Antimicrobial
Limonene	Terpenoid (Monoterpene)	Seeds, fruit	Antimicrobial, anti-inflammatory
Δ7-Avenasterol	Sterol		antimicrobial
β-Sitosterol	Sterol	Seeds, leaf	Antimicrobial
Phytosterols	Sterol	Leaf	Hepatoprotective, anti-inflammatory
Ergosta-5,22-dien-3-ol Acetate	Sterol	Root	Anti-inflammatory
Oleanolic Acid	Triterpenoid	Fruit	Anti-inflammatory, antimicrobial
2β-3β-Dihydroxy-Ursolic Acid	Triterpenoid	Leaf	Antiviral potential
Lupeol	Triterpenoid	Fruit, leaf	Antiviral potential
**OTHER BIOACTIVE COMPOUNDS**			
Linoleic Acid	Fatty Acid	Fruit peel, leaf	Antimicrobial
Saponins	Glycoside	Leaf, seeds, fruit	Antioxidant, antimicrobial
Cardioactive Glycosides	Glycoside	Leaf	Antispasmodic effect
Cardenolide	Cardiac Glycoside	Fruit	Antiviral potential
β-Mannofuranoside-Farnesyl	Glycoside	Leaf	Antiviral potential
1,8-Dichloro-9,10-Diphenylanthracene-9,10-Diol	Anthracene Derivative		Antiviral potential
Protodioscin	Steroidal Saponin	Leaf	Antiviral potential
**ENZYMES**			
Papain		Latex	Proteolytic enzyme, anti-inflammatory, antiviral potential
Chymopapain		Latex	Proteolytic enzyme, anti-inflammatory, antiviral potential
Endopeptidase Papain III and IV		Latex	Protein digestion, metabolic regulation
Glutamine Cyclotransferase		Latex	Antimicrobial
Peptidase A and B		Latex	Antimicrobial
Lysozymes		Latex	Antimicrobial

# Refer to the text for the references supporting the role of each bioactive compound during viral infection in humans.

**Table 2:** The bioactive components of papaya function as antiviral agents through various mechanisms.

Virus	Bioactive Compound	Antiviral Mechanism	References
**Chikungunya**	Papapya leaf extract; P-Coumaric Acid; Caricaxanthin; Violaxanthin; Zeaxanthin	Interfere with CHIKV replication Antiviral activity Anti-inflammatory	[69,70,71,72]
**SARS-CoV-2**	Papapya leaf extract; fermented papaya; Protodioscin; Deoxyquercetin; Kaempferol; Catechin; Apigenin; Lupeol; Carpaine; Quercetin; Kaempferol Glycosides; Phenol-2-Methyl-5-(1,2,2-Trimethylcyclopentyl)-(S)-; β-Mannofuranoside-Farnesyl; 1,8-Dichloro-9, 10-Diphenylanthracene-9,10-Diol; 2β-3β-Dihydroxy-Ursolic Acid	Anti-inflammatory and proteolytic properties Antiviral activity Inhibit SARS-CoV-2 replication Modulate cytokines (IL-6 and TNF-α) Suppress viral entry into cells by targeting the S protein and ACE2 receptor interaction	[23,58,73,74,75]
**Dengue**	Papapya leaf extract; Quercetin; Kaempferol; Carotenoids; Carpaine; Myricetin; Papain; Flavonoids; Alkaloids; Glycosides; Phenolic acids; Dehydrocarpaine I and II; Cardenolide; P-coumaric acid; Chlorogenic acid; Rutin; Caricaxanthin; Violaxanthin; Zeaxanthin; 5,7-dimethoxycoumarin; Myricetin 3-rhamnoside; Kaempferol 3-(2″-rhamnosylrutinoside)	Anti-inflammatory effects Antioxidant activity Antiviral activity Boost platelet production Inhibit DENV RNA polymerase Modulate cytokines Target viral entry	[21,23,57,58,64,66,76,77,78,79,80,81,82,83,84,85]
**Hepatitis C Virus**	Fermented papaya	Lowers pro-inflammatory cytokine Reduces oxidative stress	[86]
**Herpes Virus**	Papapya leaf extract; Apigenin	Antiviral activity	[87,88]
**HIV**	Papapya leaf extract; Kaempferol-7-glucoside; Epigallocatechin; Luteolin	Antioxidant activity Anti-HIV-1 activity Inhibit HIV-1 protease Target viral entry and replication Increase platelet count	[39,66,68,89]
**HPV**	Papapya leaf extract; Carotenoids	Antiviral activity Antioxidant activity	[90]
**Influenza**	Papapya leaf extract; P-Coumaric Acid; Chlorogenic Acid; Violaxanthin; Zeaxanthin; Quercetin; Baicalein; Oleanolic Acid	Antiviral activity Boost immune responses Inhibit viral replication Reduce oxidative stress	[69,91,92,93,94,95]
**Zika Virus**	Papapya leaf extract; Β-Sitosterol; Carpaine; Violaxanthin; Rutin; Pseudocarpaine; Δ7-Avenasterols; Cis-Β-Carotene	Anti-inflammatory activity Anti-proteolytic properties Antiviral activity Suppress viral RNA synthesis Anti-Zika replication	[19,66,96,97]
